# Radiological Evaluation of Lumbar Endplate Dimensions in the Indian Population and Their Correlation With Cage Placement/Length in Diverse Lumbar Fusion Techniques

**DOI:** 10.7759/cureus.65631

**Published:** 2024-07-29

**Authors:** Tushar Pisal, Ashwin Deshmukh, Rahul Agrawal, Sagar Gurnani, Ankit Barosani

**Affiliations:** 1 Orthopaedics, Dr. D. Y. Patil Medical College, Hospital & Research Centre, Dr. D. Y. Patil Vidyapeeth (Deemed to be University), Pune, IND

**Keywords:** interbody cages, lumbar endplates, pain, vertebral fusion, lumbar-fusion

## Abstract

Background

Lumbar fusion techniques are vital for treating various spinal conditions by promoting vertebral fusion to alleviate pain and restore stability. Given the anatomical uniqueness of the Indian skeletal structure, this study evaluates the radiological dimensions of lumbar endplates in the Indian population and their correlation with the placement and length of interbody cages used in various lumbar fusion techniques such as oblique lateral lumbar interbody fusion (OLIF), transforaminal lumbar interbody fusion (TLIF), and anterior lumbar interbody fusion (ALIF). This study aimed to conduct radiological measurements of lumbar endplates in the Indian population and correlate them with cage placement and length in lumbar fusion techniques.

Methods

This prospective study was conducted at the orthopaedic ward of a tertiary care center in Western Maharashtra, India. Healthy individuals (aged >18 years, either gender) selected with a 95% confidence level using Philip Core Integrity software (Amsterdam, Netherlands) were included in the study. We excluded those with a history of low back pain, previous lumbar spine surgeries, fractures, tuberculosis, tumors, deformities, degenerative diseases, or lesions affecting the lumbar spine. Quantitative measurements such as oblique and sagittal diameters, apophyseal ring widths, and interbody cage lengths were calculated using multiplanar reformatting with specific imaging parameters.

Results

A total of 150 individuals with an average age of 39.83 ± 14.17 years, ranging from 20 to 65 years. Among the study population, 68 were males and 82 were females. Among the male study population, oblique parameters such as Angle AOB and Mid-OD (oblique diameter) show considerable variability, with Angle AOB ranging from 51.43 ± 2.40 mm (L2 inferior) to 31.59 ± 4.25 mm (L5 inferior) and Mid-OD ranging from 41.59 ± 2.59 mm (L3 superior) to 34.38 ± 2.26 mm (S1 superior). Side-sagittal dimensions vary from 32.11 ± 2.50 mm (S1 superior) to 36.48 ±3.26 mm (L3 superior), emphasizing the need for tailored surgical planning. In contrast, females in the study population exhibit distinct anatomical profiles, with Angle AOB ranging from 52.15 ± 2.43 mm (L2 inferior) to 20.45 ± 5.45 mm (S1 superior) and Mid-OD from 33.48 ± 2.15 mm (L3 inferior) to 42.45 ± 2.59 mm (L3 superior). These findings underscore gender-specific anatomical differences crucial for individualized clinical evaluation and treatment strategies.

Conclusion

This study comprehensively analyzes oblique, side-sagittal, transverse, and midsagittal anatomic parameters across various vertebral levels in men and women, highlighting significant anatomical variations crucial for clinical assessments and surgical interventions.

## Introduction

Lumbar fusion techniques are integral to the management of a range of spinal conditions, including degenerative diseases, deformities, and instabilities [1]. The primary goal of lumbar fusion surgery is to alleviate pain and restore stability by promoting the fusion of two or more vertebrae. However, its use remains a topic of significant debate, especially for conditions such as primary disc herniation, lumbar stenosis without spondylolisthesis or deformity, and degenerative disc disease. The decision to perform lumbar fusion is multifaceted and is influenced by patient-specific factors, diagnostic findings, advancements in surgical techniques, imaging capabilities, and reimbursement policies [2]. Thus, the success of lumbar fusion hinges not only on surgical expertise but also on a comprehensive understanding of anatomical nuances, particularly those involving lumbar endplates.

Lumbar endplates, which interface between intervertebral discs and vertebral bodies, play a critical role in load transfer, spinal stability, and biomechanics [3]. These endplates are essential for ensuring proper load distribution and preventing complications such as sinking syndrome, where implants subside into the vertebral body [4]. Bone mineral density (BMD) assessments prior to surgery and strategic implant placement, particularly in the posterior-lateral regions of the endplates, are crucial for minimizing risks [5]. The upper lumbar segments, in particular, are more prone to subsidence due to decreased strength [6]. Therefore, both spine surgeons and implant designers must exercise caution when planning and executing lumbar fusion procedures.

Despite extensive global research on lumbar fusion techniques, the specific anatomical characteristics of the lumbar endplates in the Indian population have not been thoroughly explored. This population exhibits unique skeletal features, such as variations in morphology, disc dimensions, and facet joint orientation, which necessitate a tailored approach to surgical interventions [7,8]. Understanding these variations is essential for optimizing cage placement and length in lumbar fusion surgeries, thereby enhancing surgical outcomes and reducing complications.

The intricate relationship between lumbar endplate anatomy and the placement and length of interbody cages is crucial for achieving optimal surgical outcomes. Different lumbar fusion techniques, such as oblique lateral lumbar interbody fusion (OLIF), transforaminal lumbar interbody fusion (TLIF), and anterior lumbar interbody fusion (ALIF), require tailored approaches based on individual patient characteristics. This research aims to correlate radiologically measured lumbar endplate dimensions with cage placement and length in these diverse techniques, thereby ensuring optimal biomechanical support, fusion rates, and long-term stability.

## Materials and methods

This prospective study was conducted at Dr. D. Y. Patil Medical College, Pimpri, Pune, with data collected from the orthopaedic ward. Approval from the Institutional Ethics Committee was obtained, and all patients signed informed written consent before the commencement of the study. Data collection spanned from June 2022 to January 2024, involving 150 participants selected with a confidence level of 95%, calculated using Philips Core Integrity software (Amsterdam, Netherlands).

Healthy adults aged 18 years and older were included in the study, and individuals with a history of low back pain, previous lumbar spine surgeries, lumbar spine fractures, tuberculosis, tumors affecting the lumbar spine, spinal deformities, lumbar degenerative diseases, or lesions on the lumbar endplates were excluded.

Quantitative measurements

Quantitative measurements and interbody cage length calculations involved locating the vertebral pedicle origin and its projection using Picture Archiving and Communication Systems (PACS), as described previously by Sun et al. (Appendix 1) [9]. Point A represents the vertebra's center, and point O is the midpoint between the medial and lateral edges of the vertebral pedicle origin. The angle between lines OA and OB is measured as ∠AOB, and at the endplate level, point A' is the center, forming ∠A′O′B′ similarly.

Oblique diameter (Mid-OD) is defined as the distance from O' to the midpoint of the endplate at the anterior edge. Maximum-oblique diameter (Max-OD) is the longest span from point O' to the opposite side. Side-sagittal diameter (Side-SD) is measured through the left lamina's midpoint. The transverse diameter (TD) is the widest part of the endplate. Mid-sagittal diameter (Mid-SD) is measured along the endplate's bisector. The apophyseal ring widths were recorded along these diameters.

The calculations for determining the appropriate lengths of various interbody fusion cages are as follows: the minimal length of the TLIF cage is calculated as Mid-OD minus half the sum of R1 and R2. In contrast, the maximal length of the TLIF cage is Max-OD minus half the sum of R3 and R4. For the posterior lumbar interbody fusion (PLIF) cage, the length is determined by Side-SD minus half the sum of R5 and R6. The OLIF/XLIF/DLIF cage length is obtained by subtracting half the sum of R7 and R8 from the TD. Lastly, the length of the ALIF cage is calculated as Mid-SD minus half the sum of R9 and R10. These measurements ensure that the cages fit properly based on specific vertebral dimensions.

## Results

A comprehensive analysis of oblique anatomic parameters among men across various vertebral levels was done. Each parameter, including Angle AOB, Mid-OD, R1, R2, Max-OD, Max-OA, R3, and R4, was meticulously examined to identify the highest and smallest values for each parameter, highlighting anatomical variations (Table 1).

**Table 1:** Oblique anatomic parameters for men OD: oblique diameter

Vertebral Level	Angle AOB (mm)	Mid-OD (mm)	R1 (mm)	R2 (mm)	Max-OD (mm)	Max-OA (mm)	R3 (mm)	R4 (mm)
L1 inferior	50.15 ± 3.12	36.45 ± 3.15	7.45 ± 1.25	7.56 ± 1.45	42.45 ± 2.58	37.45 ± 6.89	6.47 ± 1.15	12.15 ± 2.58
L2 superior	50.43 ± 2.41	37.41 ± 2.54	7.04 ± 1.36	7.25 ± 1.48	43.84 ± 2.28	37.37 ± 6.87	5.40 ± 1.65	12.04 ± 2.26
L2 inferior	51.43 ± 2.40	40.22 ± 2.12	6.38 ± 1.45	7.48 ± 1.41	43.12 ± 2.44	35.64 ± 4.08	7.35 ± 2.11	10.37 ± 2.46
L3 superior	48.84 ± 3.07	41.59 ± 2.59	7.47 ± 1.26	6.75 ± 0.78	45.22 ± 2.35	35.09 ± 4.15	6.88 ± 0.84	9.58 ± 1.22
L3 inferior	48.45 ± 3.07	38.86 ± 2.31	7.78 ± 1.91	8.15 ± 1.48	45.54 ± 2.35	33.50 ± 5.78	11.11 ± 3.15	11.35 ± 2.99
L4 superior	44.35 ± 2.34	39.22 ± 2.79	7.58 ± 1.46	8.45 ± 1.48	46.25 ± 2.76	33.98 ± 3.43	8.81 ± 2.84	11.79 ± 1.72
L4 inferior	42.51 ± 2.34	38.23 ± 2.50	7.48 ± 1.48	7.46 ± 2.25	45.89 ± 2.41	26.89 ± 3.53	8.64 ± 1.29	12.63 ± 2.68
L5 superior	33.58 ± 4.15	37.57 ± 2.48	7.58 ± 1.79	7.00 ± 1.25	45.37 ± 3.79	20.70 ± 3.91	7.68 ± 1.98	10.39 ± 2.04
L5 inferior	31.59 ± 4.25	36.53 ± 1.90	7.65 ± 1.45	7.12 ± 1.15	46.65 ± 4.37	21.73 ± 3.19	9.15 ± 1.95	9.44 ± 2.22
S1 superior	21.74 ± 5.30	34.38 ± 2.26	7.89 ± 2.23	7.48 ± 2.17	48.15 ± 4.65	16.95 ± 2.29	10.48 ± 4.21	9.82 ± 2.0

For Angle AOB, the highest measurement was 51.43 ± 2.40 (L2 inferior), while the smallest value was 31.59 ± 4.25 (L5 inferior). The Mid-OD parameter showed the highest value at 41.59 ±2.59 (L3 superior) and the smallest at 34.38 ± 2.26 (S1 superior). R1 exhibits the widest range, with the highest measurement at 7.89 ± 2.23 (S1 superior) and the smallest at 6.38 ±1.45 (L2 inferior). R2 exhibited significant differences, with the highest value at 8.45 ± 1.48 (L4 superior) and the smallest at 6.75 ± 0.78 (L3 superior).

For Max-OD, the largest recorded measurement was 48.15 ± 4.65 (S1 superior), while the smallest was 42.45 ± 2.58 (L1 inferior). Max-OA varies notably, with the highest value at 37.45± 6.89 (L1 inferior) and the smallest at 16.95 ± 2.29 (S1 superior). R3 ranges from 11.11 ±3.15 (L3 inferior) to 5.40 ± 1.65 (L2 superior), and R4 from 12.63 ± 2.68 (L4 inferior) to 9.44± 2.22 (L5 inferior).

The side-sagittal anatomic parameters for men across various vertebral levels, specifically focusing on the Side-SD and the widths of the apophyseal ring (R5 and R6), were analyzed. The Side-SD varied from 32.11 ± 2.50 mm at the S1 superior level to 36.48 ± 3.26 mm at the L3 superior level. R5 showed its highest measurement of 7.19 ± 1.69 mm at the L3 inferior level and the lowest at 5.80 ± 1.76 mm at the S1 superior level. Similarly, R6 ranges from a maximum of 7.48 ± 1.65 mm at the L1 inferior level to a minimum of 6.17 ±1.29 mm at the S1 superior level. These parameters highlight the anatomical variations that are crucial for tailoring surgical interventions in lumbar fusion procedures, emphasizing the need for precise measurement and individualized planning to optimize surgical outcomes (Table 2).

**Table 2:** Side-sagittal anatomic parameters for men SD: sagittal diameter

Side-Sagittal	Side-SD (mm)	R5 (mm)	R6 (mm)
L1 inferior	36.45 ± 2.45	6.78 ± 1.45	7.48 ± 1.65
L2 superior	35.27 ± 2.68	6.83 ± 1.49	7.15 ± 1.47
L2 inferior	35.34 ± 2.48	6.65 ± 1.27	6.48 ± 1.63
L3 superior	36.48 ± 3.26	7.08 ± 1.37	6.78 ± 0.87
L3 inferior	35.70 ± 2.66	7.19 ± 1.69	6.45 ± 1.38
L4 superior	36.24 ± 3.37	6.68 ± 1.29	6.95 ± 1.50
L4 inferior	35.36 ± 2.73	7.02 ± 1.66	7.30 ± 1.88
L5 superior	35.10 ± 2.62	5.85 ± 1.22	6.93 ± 1.57
L5 inferior	34.46 ± 3.35	6.58 ± 1.78	6.95 ± 1.27
S1 superior	32.11 ± 2.50	5.80 ± 1.76	6.17 ± 1.29

Oblique anatomic parameters were assessed for females, detailing various measurements across different vertebral levels (Table 3). The Angle AOB (mm) ranged from 20.45 ± 5.45 mm at the S1 superior level to 52.15 ± 2.43 mm at the L2 inferior level, indicating significant variability in the oblique angle. The Mid-OD (mm) shows a range from 33.48 ± 2.15 mm at the L3 inferior level to 42.45 ± 2.59 mm at the L3 superior level. The parameters R1 and R2 also demonstrated variations, with R1 ranging from 6.39 ± 1.55 mm at L2 inferior to 8.45 ± 2.15 mm at L5 superior and R2 ranging from 6.12 ± 1.15 mm at L5 inferior to 8.25 ± 1.88 mm at L2 superior. The Max-OD (mm) varied between 41.15 ± 2.48 mm at L1 inferior and 49.55 ± 4.55 mm at S1 superior, while Max-OA (mm) ranges from 15.45 ± 2.25 mm at S1 superior to 38.45 ± 6.59 mm at L1 inferior. The R3 and R4 parameters showed their highest values at L3 inferior (11.11± 3.15 mm) and L4 inferior (13.64 ± 2.58 mm), respectively, and their lowest at L3 superior (0.88 ± 0.84 mm) and S1 superior (8.82 ± 1.09 mm), respectively. These data points underscore the anatomical differences at each vertebral level, which are critical for individualized clinical assessments and interventions in women.

**Table 3:** Oblique anatomic parameters for females OD: oblique diameter

Oblique Female	Angle AOB (mm)	Mid-OD (mm)	R1 (mm)	R2 (mm)	Max-OD (mm)	Max-OA (mm)	R3 (mm)	R4 (mm)
L1 inferior	49.12 ± 2.45	35.15 ± 2.95	7.15 ± 1.25	6.46 ± 1.25	41.15 ± 2.48	38.45 ± 6.59	6.48 ± 1.15	11.15 ± 1.58
L2 superior	48.51 ± 2.45	37.45 ± 2.48	7.04 ± 1.56	8.25 ± 1.88	44.64 ± 2.28	36.54 ± 6.87	5.41 ± 1.65	11.04 ± 2.26
L2 inferior	52.15 ± 2.43	41.45 ± 2.11	6.39 ± 1.55	7.48 ± 1.61	42.22 ± 2.44	34.64 ± 4.08	6.35 ± 2.11	11.87 ± 2.46
L3 superior	47.45 ± 3.25	42.45 ± 2.59	7.45 ± 1.24	6.75 ± 0.78	44.12 ± 2.15	34.01 ± 4.55	0.88 ± 0.84	9.15 ± 1.22
L3 inferior	47.95 ± 3.07	33.48 ± 2.15	7.45 ± 1.90	7.25 ± 1.78	44.64 ± 2.35	34.60 ± 5.78	11.11 ± 3.15	10.36 ± 2.59
L4 superior	43.56 ± 2.45	38.15 ± 2.78	8.15 ± 1.47	8.15 ± 1.68	45.55 ± 2.66	34.91 ± 3.53	8.81 ± 2.84	12.75 ± 1.72
L4 inferior	43.45 ± 2.35	39.15 ± 2.48	8.26 ± 1.48	7.66 ± 2.25	44.69 ± 2.61	25.48 ± 3.43	8.64 ± 1.29	13.64 ± 2.58
L5 superior	34.54 ± 4.14	38.48 ± 2.49	8.45 ± 2.15	7.10 ± 1.25	46.35 ± 3.89	21.40 ± 3.91	8.68 ± 1.98	11.59 ± 2.04
L5 inferior	30.58 ± 3.59	37.65 ± 1.90	8.45 ± 1.45	6.12 ± 1.15	47.65 ± 4.87	20.72 ± 3.14	8.15 ± 1.95	9.54 ± 2.22
S1 superior	20.45 ± 5.45	34.58 ± 2.26	7.87 ± 2.23	6.58 ± 1.17	49.55 ± 4.55	15.45 ± 2.25	9.48 ± 4.61	8.82 ± 1.09

Further, the side-sagittal anatomic parameters for females, including measurements across various vertebral levels, were analyzed (Table 4). The Side-SD (mm) parameter varied from 29.16 ± 2.45 mm at the L5 inferior level to 36.46 ± 2.43 mm at the L4 inferior level, indicating the breadth of side-sagittal dimensions. The R5 (mm) parameter showed its lowest value at the L2 inferior level (5.15 ± 1.27 mm) and its highest at the L4 superior level (8.69 ± 3.29 mm). Similarly, R6 (mm) ranged from 4.26 ± 1.25 mm at L3 inferior to 6.25 ± 1.41 mm at L2 inferior. These variations highlight the anatomical differences along the spinal column, which are essential for precise clinical evaluation and treatment planning for females.

**Table 4:** Side-sagittal anatomic parameters for females SD: sagittal diameter

Side-Sagittal	Side- SD (mm)	R5 (mm)	R6 (mm)
L1 inferior	30.12 ± 1.55	5.78 ± 1.45	4.58 ± 1.45
L2 superior	30.57 ± 1.18	5.23 ± 1.49	5.85 ± 1.45
L2 inferior	32.12 ± 1.88	5.15 ± 1.27	6.25 ± 1.41
L3 superior	30.15 ± 2.16	7.09 ± 1.37	5.15 ± 0.56
L3 inferior	30.10 ± 1.76	6.11 ± 2.69	4.26 ± 1.25
L4 superior	29.54 ± 3.47	8.69 ± 3.29	5.36 ± 1.60
L4 inferior	36.46 ± 2.43	7.15 ± 2.66	6.25 ± 1.25
L5 superior	30.11 ± 2.12	6.78 ± 1.22	6.26 ± 1.65
L5 inferior	29.16 ± 2.45	7.15 ± 2.78	6.22 ± 1.25
S1 superior	30.10 ± 2.40	6.15 ± 2.76	5.44 ± 1.59

The transverse and midsagittal anatomic parameters for men across various vertebral levels were measured (Table 5). The TD ranges from 48.08 ± 2.45 mm at L2 superior to 53.37 ± 2.23 mm at L4 inferior. The R7 parameter, representing a specific anatomic measurement, exhibits considerable variation, with the lowest value at L1 inferior (4.14 ± 1.52 mm) and the highest at L3 inferior (11.45 ± 2.72 mm). Similarly, R8 ranges from 5.25 ± 1.36 mm at L3 superior to 9.65 ± 2.12 mm at L5 inferior. The Mid-SD varies between 33.73 ± 2.65 mm at L1 inferior and 36.38 ± 2.65 mm at L2 inferior. R9 and R10 parameters, which are additional specific measurements, also show variability, with R9 ranging from 5.58 ± 2.31 mm at L5 inferior to 7.52 ± 1.53 mm at L4 superior and R10 ranging from 5.64 ± 0.71 mm at L3 superior to 7.29 ± 1.62 mm at L5 inferior. These variations highlight the anatomical differences along the spinal column, which are crucial for precise clinical assessment and treatment planning.

**Table 5:** Transverse and midsagittal anatomic parameters for men TD: transverse diameter; SD: sagittal diameter

Vertebral Level	R7 (mm)	R8 (mm)	TD (mm)	R9 (mm)	R10 (mm)	Mid-SD (mm)
L1 inferior	4.14 ± 1.52	7.48 ± 1.15	48.87 ±2.66	7.05 ± 1.15	6.88 ± 1.19	33.73 ±2.65
L2 superior	7.28 ± 1.23	7.54 ± 0.73	48.08 ±2.45	6.39 ± 1.07	6.94 ± 1.26	34.83 ±2.36
L2 inferior	9.45 ± 2.15	6.45 ± 1.65	48.13 ±2.59	6.28 ± 1.45	6.55 ± 1.49	36.38 ±2.65
L3 superior	5.89 ± 1.29	5.25 ± 1.36	48.90 ±3.14	6.32 ± 0.98	5.64 ± 0.71	34.96 ±2.25
L3 inferior	11.45 ±2.72	8.69 ± 2.35	50.14 ±3.61	6.22 ± 1.55	6.74 ± 1.61	35.08 ±2.45
L4 superior	8.12 ± 1.66	7.26 ± 1.59	51.16 ±3.22	7.52 ± 1.53	7.19 ± 1.52	35.78 ±3.15
L4 inferior	10.73 ±1.75	8.11 ± 2.54	53.37 ±2.23	6.03 ± 1.45	6.45 ± 1.34	35.18 ±2.66
L5 superior	9.24 ± 3.30	6.52 ± 1.58	50.85 ±3.89	6.78 ± 6.52	6.65 ± 1.53	36.05 ±1.28
L5 inferior	8.95 ± 1.41	9.65 ± 2.12	51.77 ±3.51	5.58 ± 2.31	7.29 ± 1.62	34.12 ±2.40
S1 superior	9.67 ± 3.28	9.36 ± 3.01	48.10 ±6.07	6.79 ± 1.22	6.15 ± 1.04	33.83 ±1.81

Transverse and midsagittal anatomic parameters for women across various vertebral levels are shown in Table 6. The TD varies from 40.55 ± 2.56 mm at L3 superior to 48.57 ± 3.15 mm at L5 inferior. The R7 parameter showed the lowest value at L1 inferior (5.28± 1.15 mm) and the highest at L4 inferior (10.65 ± 1.84 mm). Similarly, R8 ranges from 5.49± 0.45 mm at L3 superior to 9.55 ± 1.87 mm at L5 inferior. The Mid-SD spans from 27.52 ± 1.65 mm at L2 superior to 30.44 ± 1.41 mm at L5 inferior. The R9 and R10 parameters showed considerable variation, with R9 ranging from 5.23 ± 1.15 mm at L2 inferior to 7.65 ± 1.65 mm at L5 inferior and R10 ranging from 4.65 ± 0.69 mm at L4 inferior to 5.58± 0.45 mm at L5 superior. These data illustrate the anatomical differences along the spinal column in women, which is important for clinical evaluations and treatments.

**Table 6:** Transverse and midsagittal anatomic parameters for women TD: transverse diameter; SD: sagittal diameter

Vertebral Level	Transverse			Midsagittal		
	R8 (mm)	TD (mm)	R7 (mm)	R9 (mm)	R10 (mm)	Mid-SD (mm)
L1 inferior	6.56 ± 1.16	40.72 ±2.68	5.28 ± 1.15	6.52 ± 1.28	5.28 ± 1.65	28.79 ±1.45
L2 superior	6.09 ± 0.48	41.25 ±2.78	5.48 ± 0.45	6.38 ± 1.25	4.98 ± 1.15	27.52 ±1.65
L2 inferior	6.65 ± 0.88	41.11 ±2.48	7.28 ± 0.89	5.23 ± 1.15	4.65 ± 0.78	28.80 ±1.15
L3 superior	5.49 ± 0.45	40.55 ±2.56	6.54 ± 1.45	6.15 ± 0.69	5.15 ± 1.45	29.33 ±1.25
L3 inferior	7.24 ± 1.25	47.56 ±2.48	8.25 ± 1.46	7.15 ± 0.48	5.18 ± 1.98	28.77 ±1.48
L4 superior	6.81 ± 1.22	45.65 ±3.65	6.65 ± 1.33	6.10 ± 0.65	5.18 ± 0.89	28.58 ±1.78
L4 inferior	9.45 ± 1.28	46.56 ±2.26	10.65 ±1.84	7.15 ± 1.87	4.65 ± 0.69	30.03 ±2.15
L5 superior	8.03 ± 1.21	47.69 ±2.35	7.65 ± 1.58	6.48 ± 0.48	5.58 ± 0.45	29.75 ±1.45
L5 inferior	9.55 ± 1.87	48.57 ±3.15	10.65 ±1.45	7.65 ± 1.65	5.54 ± 1.65	30.44 ±1.41
S1 superior	7.89 ± 1.02	48.16 ±3.48	9.48 ± 2.18	6.48 ± 1.68	5.25 ± 1.54	29.74 ±1.57

A comparison of corresponding anatomic parameters for men across various vertebral levels, specifically L1/2, L2/3, L3/4, L4/5, and L5/S1 was done. These parameters include Mid-OD, Max-OD, Max-OA, Side-SD, TD, and Mid-SD (Table 7). Notably, L1/2 showed high values for Mid-OD, Max-OD, and Max-OA, indicating a larger dimension at this level compared to others. Conversely, L2/3 exhibited a significant Max-OD but lower values for other parameters, suggesting variability in its anatomical structure. L3/4 showed moderate values across most parameters, with Side-SD being relatively higher, while L4/5 displayed high Side-SD and Mid-SD, indicating greater dimensions in these regions. Finally, L5/S1 had lower values for all parameters, particularly Max-OA and Mid-SD, highlighting its distinct anatomical characteristics. This table underscores the variations in spinal dimensions, which are essential for clinical assessments and treatments tailored to different spinal levels in men.

**Table 7:** Comparison of corresponding anatomic parameters for men OD: oblique diameter; SD: sagittal diameter; TD: transverse diameter

Parameter (Men)	Mid-OD	Max-OD	Max-OA	Side-SD	TD	Mid-SD
L1/2	0.6512	0.9178	0.9758	0.0001	0.003	0.0020
L2/3	0.2145	0.8445	0.0654	0.0152	0.0515	0.2325
L3/4	0.2585	0.1945	0.4256	0.3645	0.1758	0.1115
L4/5	0.1754	0.5645	0.5845	0.4565	0.0035	0.7515
L5/S1	0.0654	0.0654	0.0265	0.0354	0.1765	0.0452

Further, a detailed comparison of corresponding anatomic parameters for women at various vertebral levels, namely L1/2, L2/3, L3/4, L4/5, and L5/S1, was conducted. The parameters measured include Mid-OD, Max-OD, Max-OA, Side-SD, TD, and Mid-SD (Table 8). The data reveal that L1/2 had a very low Mid-OD but high Max-OD, indicating a significant difference in dimensions. For L2/3, Max-OD was moderately high, but other parameters were relatively low, highlighting less variation. The L3/4 level showed significant Mid-SD and moderate values for other parameters, suggesting considerable anatomical variability. L4/5 stood out with a high Max-OA and Mid-SD, indicating larger dimensions in these areas. Lastly, L5/S1 exhibited high values for Side-SD and TD, suggesting a distinct anatomical structure at this level. This comparison underscores the variability in spinal anatomy among women, which is crucial for clinical assessments and interventions specific to different spinal regions.

**Table 8:** Comparison of corresponding anatomic parameters for women OD: oblique diameter; SD: sagittal diameter; TD: transverse diameter

Parameter (Women)	Mid-OD	Max-OD	Max-OA	Side-SD	TD	Mid-SD
L1/2	0.0061	0.9448	0.0025	0.0025	0.0029	0.0565
L2/3	0.0544	0.5465	0.0845	0.0854	0.0015	0.0854
L3/4	0.5895	0.0035	0.4585	0.0345	0.0452	0.6815
L4/5	0.2658	0.3954	0.7545	0.0014	0.0014	0.5361
L5/S1	0.0212	0.6541	0.0058	0.5845	0.5854	0.0145

The recommended lengths for different types of lumbar interbody fusion procedures, which are surgical techniques used to treat spinal conditions by fusing vertebrae together using implants, are presented in Table 9. For minimally invasive TLIF (Min-TLIF), the recommended cage lengths ranged from 26.45 mm at the L5/S1 level to 36.12 mm at L2/3. This approach typically involved accessing the spine through a small incision and inserting the cage through the intervertebral foramen. Max-TLIF refers to the maximum advised cage lengths for TLIF, ranging from 35.12 mm at L1/2 to 38.15 mm at L4/5, which may be used when more extensive support or correction is required. PLIF involves recommended cage lengths ranging from 26.12 mm at L5/S1 to 30.45 mm at L2/3, accessed from a posterior approach. Oblique, extreme, or direct lateral lumbar interbody fusion (OLIF/XLIF/DLIF) techniques recommend cage lengths from 39.45 mm at L1/2 to 43.15 mm at L5/S1, accessed laterally, which avoids significant disruption to the back muscles. ALIF utilizes recommended cage lengths ranging from 28.15 mm at L3/4 to 29.45 mm at L4/5, accessed through an anterior approach, often providing good access to the lumbar spine without disruption of the spinal muscles. These lengths are crucial as they ensure adequate support and fusion between vertebrae, promoting spinal stability and alleviating symptoms caused by spinal disorders like degenerative disc disease or instability. The choice of procedure and cage length depends on the specific spinal condition, patient anatomy, and surgical goals.

**Table 9:** Advised length of cages using different approaches for men TLIF: transforaminal lumbar interbody fusion; PLIF: posterior lumbar interbody fusion; OLIF: oblique lateral lumbar interbody fusion; XLIF: extreme lumbar interbody fusion; DLIF: direct lateral lumbar interbody fusion; ALIF: anterior lumbar interbody fusion

Parameter (Men)	Min-TLIF (mm)	Max-TILF (mm)	PLIF (mm)	OLIF/XLIF/DLIF (mm)	ALIF (mm)
L1/2	32.25	35.12	29.36	39.45	28.45
L2/3	36.12	36.15	30.45	42.15	28.26
L3/4	31.25	36.48	29.65	42.56	28.15
L4/5	30.25	38.15	28.15	42.56	29.45
L5/S1	26.45	36.92	26.12	43.15	28.45

Table 10 presents the recommended cage lengths for various lumbar interbody fusion techniques tailored for female patients. Each technique (Min-TLIF, Max-TLIF, PLIF, OLIF/XLIF/DLIF, and ALIF) is associated with specific cage lengths across different lumbar spinal levels (L1/2 to L5/S1). For instance, Min-TLIF ranges from 24.12 mm at L5/S1 to 29.12 mm at L2/3, indicating minimal invasiveness and targeted spinal stabilization through small incisions. Conversely, techniques such as ALIF recommend lengths from 23.45 mm at L1/2 to 26.25 mm at L4/5, involving anterior access for direct disc management. These guidelines are crucial for surgeons in selecting the appropriate procedure and implant size based on individual patient anatomy and clinical needs, ensuring effective spinal fusion and postoperative outcomes in female patients.

**Table 10:** Advised length of cages using different approaches for women TLIF: transforaminal lumbar interbody fusion; PLIF: posterior lumbar interbody fusion; OLIF: oblique lateral lumbar interbody fusion; XLIF: extreme lumbar interbody fusion; DLIF: direct lateral lumbar interbody fusion; ALIF: anterior lumbar interbody fusion

Parameter	Min-TLIF (mm)	Max-TLIF (mm)	PLIF (mm)	OLIF/XLIF/DLIF (mm)	ALIF (mm)
L1/2	26.45	29.54	25.54	36.02	23.45
L2/3	29.12	30.45	26.15	36.25	25.15
L3/4	28.65	33.15	25.78	38.45	25.65
L4/5	25.66	32.58	26.02	29.65	26.25
L5/S1	24.12	33.56	21.25	N/A	24.12

## Discussion

The study delves into the variability of endplate dimensions within the Indian population, underscoring the necessity for tailored considerations in spinal surgery. It highlights how different lumbar fusion techniques, such as OLIF, TLIF, and ALIF, require distinct approaches due to anatomical differences across populations. Specifically, OLIF, which accesses the spine laterally, must navigate through varying endplate morphologies that differ from those encountered in TLIF or ALIF procedures [10]. This understanding is crucial for surgeons in determining optimal cage placement and length, which is essential for achieving stable spinal fusion and reducing postoperative complications. Due to the composition of cancellous and cortical bone, which varies across the vertebral endplates, researchers have extensively studied their biomechanical properties.

Grant et al. (2001) conducted a study using cadaveric specimens to explore these characteristics through indentation tests specifically focused on the lower lumbar and sacral regions [11]. Their findings highlighted significant differences in strength and stiffness across different areas of the endplates. Particularly, the outer regions, notably the posterolateral area, were identified as stronger and stiffer compared to the central region (P < 0.0001). Additionally, the study revealed disparities between superior and inferior lumbar endplates, with the inferior endplates exhibiting significantly greater mechanical properties in terms of failure load (P = 0.0080) and stiffness (P = 0.0027). These findings underscore the heterogeneous nature of vertebral endplates and provide critical insights into their structural variability, crucial for understanding spinal biomechanics and guiding surgical approaches aimed at enhancing spinal stability and patient outcomes [11].

Our study revealed significant variations in several key anatomical parameters of the vertebral column. Measurements such as Angle AOB ranged widely from 51.43 ± 2.40 degrees at L2 inferior to 31.59 ± 4.25 degrees at L5 inferior, demonstrating diverse spinal curvature across different levels. Additionally, parameters such as Mid-OD showed variations from 41.59 ± 2.59 mm at L3 superior to 34.38 ± 2.26 mm at S1 superior, indicating differences in vertebral diameter along the oblique plane. The width of the apophyseal ring (R1) ranged from 7.89 ±2.23 mm at S1 superior to 6.38 ± 1.45 mm at L2 inferior, underscoring anatomical diversity. These findings highlight the complexity and variability in vertebral morphology, which is crucial for surgical planning and understanding spinal biomechanics. Furthermore, in procedures such as OLIF, these anatomical nuances are pivotal in optimizing surgical outcomes by ensuring adequate access through the natural operating window between the psoas major muscle and the abdominal structures.

In a study conducted by Han et al. (2022), the width of the surgical operating window for OLIF was defined as the shortest distance between the left psoas major muscle and the abdominal aorta [12]. Measurements at intervertebral spaces L2-3, L3-4, and L4-5 were reported as 16.25 ± 4.22 mm, 15.46 ± 4.64 mm, and 11.71 ± 6.29 mm, respectively, highlighting variations in surgical access based on vertebral level. Davis et al., in their autopsy study on OLIF, corroborated these findings with access corridor diameters of 18.60 mm at L2-3, 19.25 mm at L3-4, and 15.00 mm at L4-5 under static conditions, closely aligning with the parameters of the study. Furthermore, Pearson correlation analysis indicated a negative correlation (r = -0.337, P < 0.001) between surgical window width and intervertebral space, indicating narrower windows in lower vertebrae. While women generally showed slightly larger dimensions than men at the L3-4 level, these gender differences were not statistically significant across other vertebral segments. These findings underscore the anatomical variability and gender considerations crucial in optimizing surgical approaches and outcomes in OLIF procedures.

Lowe et al. conducted an in vitro study focusing on the regional strength of vertebral endplates, aiming to optimize the geometry and cross-sectional area for structural interbody support and endplate preparation. Their findings, derived from indentation tests, indicated that the central and anterocentral regions of endplates exhibit lower resistance to subsidence, highlighting vulnerabilities in these areas when considering surgical implantations. Building on this foundation, ongoing research into cage designs continues to evolve. Matsumura et al. (2006) conducted a retrospective analysis comparing postoperative subsidence rates between different cages used in TLIF/PLIF procedures. They theorized that cages with longer anteroposterior diameters and larger cross-sectional areas would enhance load-bearing capabilities, potentially reducing the incidence of subsidence [13].

Tohmeh et al. (2014) [14] performed a prospective study on cage settling rates following XLIF, revealing that 50-mm cages were more prone to severe subsidence compared to 60-mm cages. Additionally, cages with lengths of 45 mm and 55 mm showed higher rates of subsidence compared to the 60-mm counterparts. These findings suggest that using longer cages may mitigate subsidence by providing broader coverage across the peripheral region of the endplate, thereby distributing loads more effectively. Faizan et al. (2014) employed biomechanical finite element modeling to compare an articulating vertebral interbody device with a conventional TLIF device [15]. Their study concluded that larger and longer TLIF devices offer superior load-sharing capabilities and more homogeneous stress distribution across the peripheral region of the endplate. This research underscores the critical role of the peripheral region in ensuring the stability and longevity of spinal fusion constructs. Overall, these studies advocate for the adoption of larger and longer cages in spinal fusion surgeries to optimize load distribution, reduce subsidence risks, and improve overall clinical outcomes.

Various imaging modalities, including plain film radiography, CT, and MRI, provide detailed cross-sectional images that facilitate precise measurement of anatomical structures, making them highly relevant for this research. MRI offers high-quality, non-ionizing, three-dimensional images of the lumbar spine, although current guidelines for its use are somewhat unclear. This study also leverages advanced imaging techniques, particularly CT, to conduct a comprehensive radiological assessment of lumbar endplate dimensions in the Indian population.

Although our study contributes significantly to the field, however, we acknowledge certain limitations. The focus on anatomical variability in vertebral endplate dimensions does not extend to long-term clinical outcomes or postoperative complications associated with different spinal fusion techniques. By not addressing how these anatomical differences influence the success rates, complications, or recovery times of various fusion methods, the study misses an opportunity to provide a more comprehensive understanding of how anatomical variability impacts surgical results. Including such data could offer valuable insights into the practical implications of anatomical differences, potentially guiding more effective surgical planning and improving patient outcomes in spinal surgery.

## Conclusions

This work highlighted the significance of radiological measurements of endplates among the Indian population and their correlation with cage placement and length in lumbar fusion techniques such as OLIF, TLIF, and ALIF. By elucidating anatomical variations specific to this demographic, the study underscores the critical role of understanding these variations in planning successful spinal surgeries. The findings emphasize the need for personalized approaches in cage selection and placement, aiming to optimize spinal alignment, stability, and fusion outcomes while minimizing complications. This work not only informs clinical practice but also lays the groundwork for future studies and the development of tailored guidelines in spinal surgery for the Indian context, ultimately enhancing surgical precision and patient outcomes.

Understanding the correlation between lumbar endplate anatomy and the choice of fusion technique is pivotal for developing patient-specific treatment strategies. By minimizing the risk of complications and enhancing surgical outcomes, this research endeavors to provide valuable insights that refine surgical strategies to be both clinically precise and culturally sensitive. The ultimate goal is to contribute to advancements in spinal surgery that are tailored to the anatomical and cultural specificities of the Indian population, thereby improving long-term patient satisfaction and success rates in lumbar fusion procedures.

## Figures and Tables

**Figure 1:**
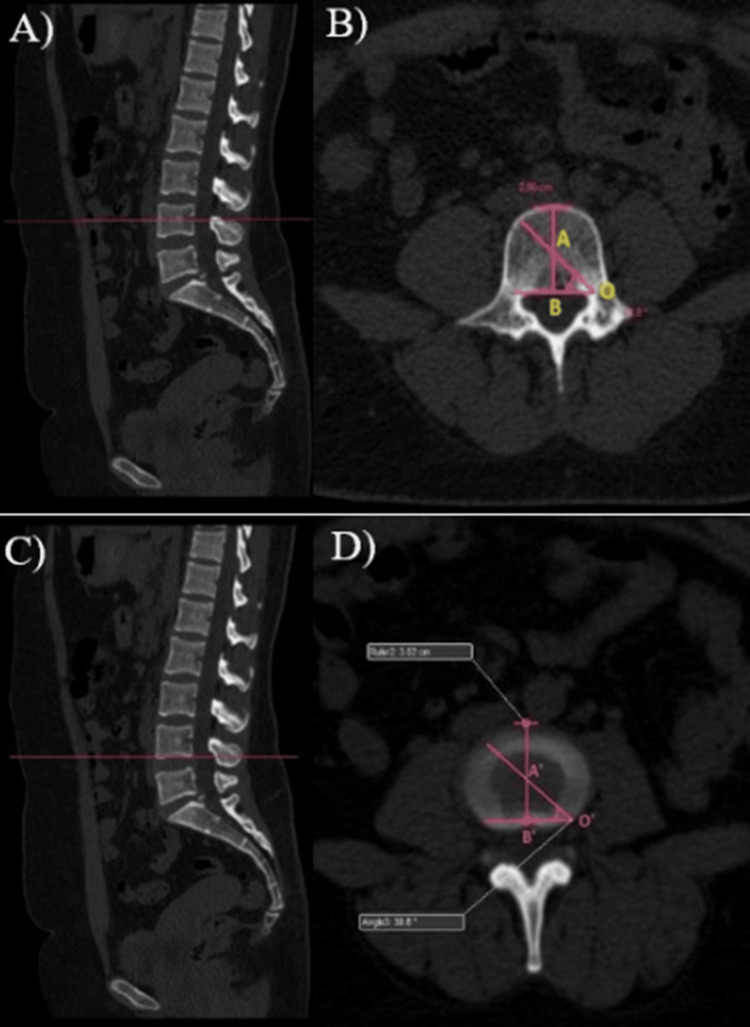
Vertebral pedicle origin and its projection

**Figure 2:**
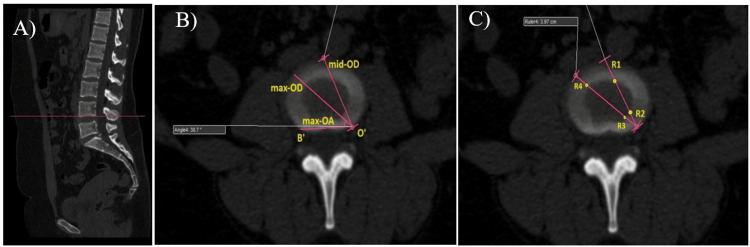
OD and width of the apophyseal ring OD: oblique diameter

**Figure 3:**
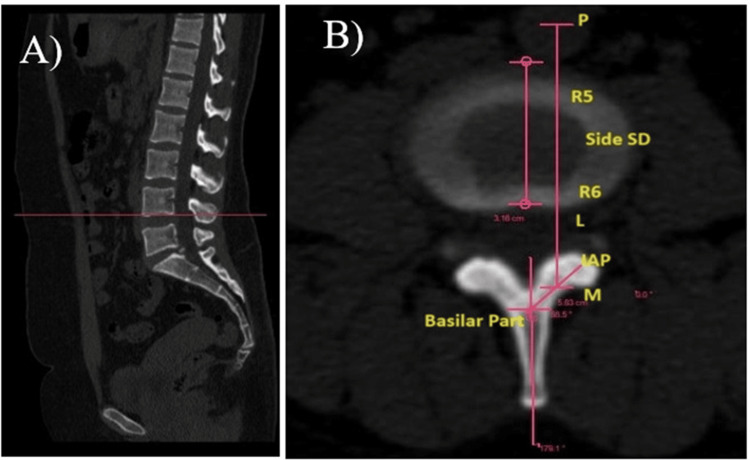
Side-SD and width of the apophyseal ring Side-SD: side-sagittal diameter

**Figure 4:**
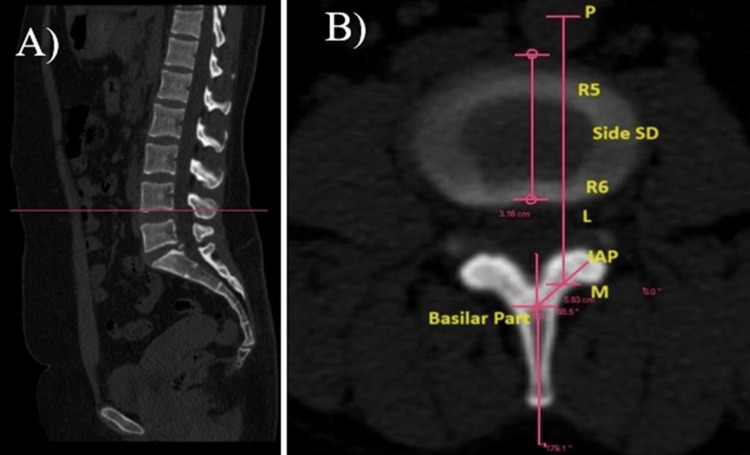
TD, Mid-SD, and width of the apophyseal ring TD: transverse diameter; Mid-SD: mid-sagittal diameter

## Appendices

Quantitative measurements and interbody cage length were done according to the following parameters (Figures 1, 2, 3, 4).
